# Supplementary material for: The anti-obesogenic metabolite, Lac-Phe, is elevated by metformin treatment in prostate cancer patients
Source: EMBO Mol Med. 2026 Apr 6;18(5):1551–62. doi: 10.1038/s44321-026-00408-6 (PMC13179383; doi:10.1038/s44321-026-00408-6)
Supplement: Supplementary file 1 — Appendix [file 44321_2026_408_MOESM1_ESM.pdf]

Table of contents

- **Appendix Figure S1A** **Page 1/3**
  Chromatogram displaying the retention time of Lac-Phe in a serum sample (green trace) compared to the authentic chemical standard (red trace).
- **Appendix Figure S1B** **Page 2/3**
  Mass spectrometry results showing the quantifier ion at m/z 88.1 and the qualifier ion at m/z 91.0 for Lac-Phe.
- **Appendix Figure S1C** **Page 3/3**
  Standard curve for calculating absolute values of Lac-Phe.

Appendix Figure S1A

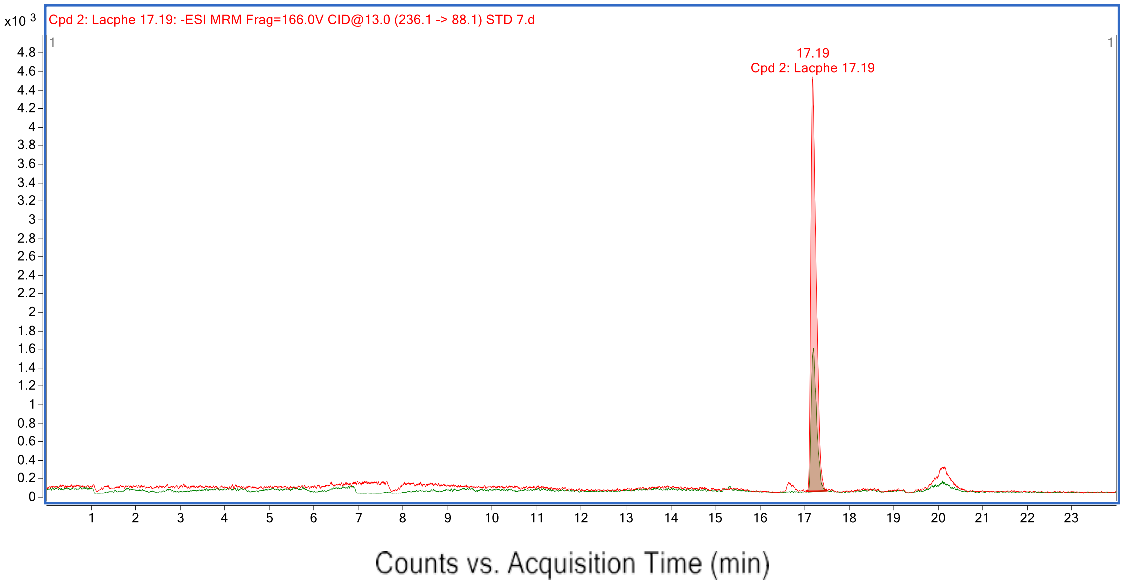

Appendix Figure S1A: Chromatogram displaying the retention time of Lac-Phe in a serum sample (green trace) compared to the authentic chemical standard (red trace).

Appendix Figure S1B

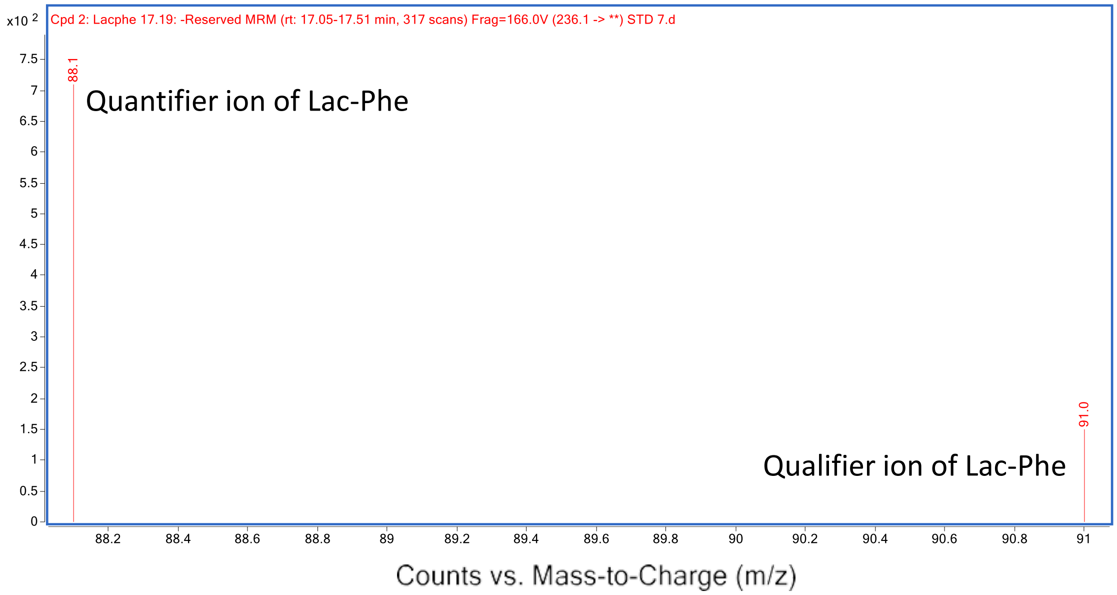

Appendix Figure S1B: Mass spectrometry results showing the quantifier ion at m/z 88.1 and the qualifier ion at m/z 91.0 for Lac-Phe.

Appendix Figure S1C

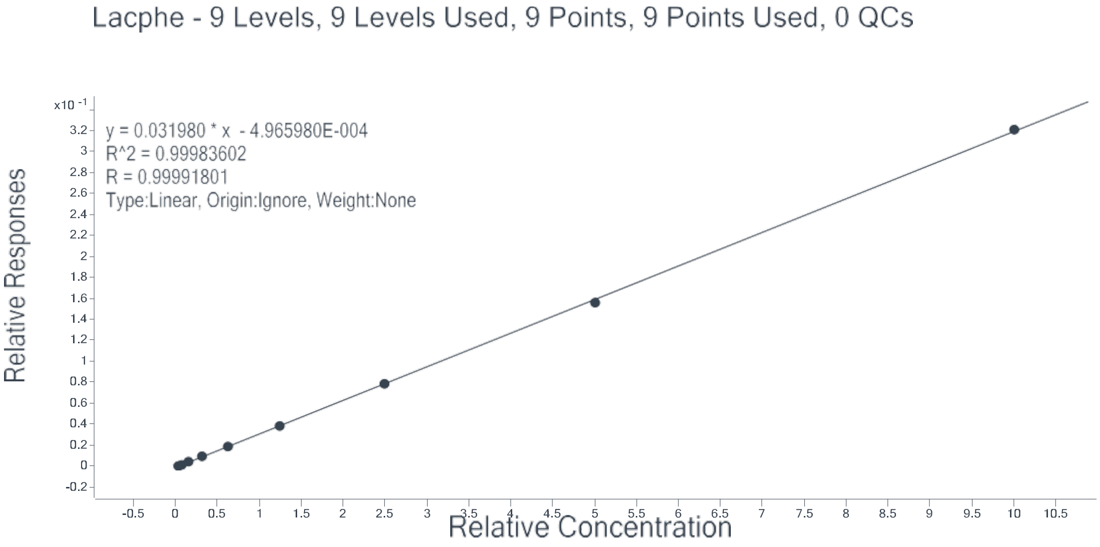

Appendix Figure S1C: Standard curve for calculating absolute values of Lac-Phe.
