# Supplementary material for: The anti-obesogenic metabolite, Lac-Phe, is elevated by metformin treatment in prostate cancer patients
Source: EMBO Mol Med. 2026 Apr 6;18(5):1551–62. doi: 10.1038/s44321-026-00408-6 (PMC13179383; doi:10.1038/s44321-026-00408-6)
Supplement: Supplementary file 7 — Expanded View Figures [file 44321_2026_408_MOESM7_ESM.pdf]

Expanded View Figures

Figure EV1. Information for patient cohorts from the BIMET-1 trial and from the University of Miami/Sylvester Comprehensive Cancer Center's umbrella biomarker protocol who were profiled in this study.

(**A**) Age, PSA levels and BMI for the patients ($n = 5$) in Arm A and patients ($n = 7$) in Arm B from the BIMET-1 trial. The patients in Arm B are further subdivided into responders, R (in red), or non-responders, NR. Positive response was defined as a decline in serum PSA. *One patient was categorized as a partial responder due to a rapid decrease in PSA for the first four weeks of metformin monotherapy but then followed by an increasing PSA. **Patient sera samples that were unavailable at the end of D225. (**B**) Trendlines showing changes in lactate at the D56 timepoint vs. D1 for individual Arm B patients from the BIMET-1 trial who were classified as metformin responders (R) or non-responders (NR). (**C**) Disease stage, age, BMI, PSA levels, and hormone therapy status for prostate cancer patients enrolled through an umbrella biomarker specimen collection protocol and profiled in this study for Lac-Phe levels in Fig. 1F. Source data are available online for this figure.

© The Author(s)

A

Patient ID	Days of blood collection	Age	Gleason	Baseline PSA	PSA (56d)	PSA (225d)	Arm	Met Response (PSA decline 56d)	BMI (baseline)	BMI (56d)	BMI (225d)
B-2	1, 56, 225	67	4+3=7	3.72	**3.53**	0.02	metformin	**R**	**28.3**	**28.1**	**28.1**
B-9	1, 56, 225	64	4+4=8	5.41	**5.2**	0.36	metformin	**R**	**29.9**	**29.1**	**29.2**
B-12	1, 56, 225	53	4+5=9	2.09	**1.88**	0.7	metformin	**R**	**27.1**	**24.2**	**25.7**
B-16*	1, 56	72	4+3=7	0.88	0.93	0.3	metformin	R*	28.4	28.3	25.8**
B-3	1, 56, 225	74	3+5=8	16.26	20.22	2.14	metformin	NR	25.6	25.4	26.3
B-14	1, 56, 225	58	4+5=9	4.07	5.1	0.14	metformin	NR	30.4	29.8	29.5
B-15	1, 56, 225	73	4+3=7	10.76	13.1	0.66	metformin	NR	25.9	25.4	25.1**
A-1	1, 56, 225	63	4+3=7	1.18	1.53	0.06	observation	NR	33.1	33.6	35
A-6	1, 56, 225	68	3+4=7	10.99	16.23	2.8	observation	NR	29.4	30.1	30
A-8	1, 56, 225	60	5+4=9	7.48	8.27	1.49	observation	NR	33.8	33.2	33.1
A-10	1, 56, 225	56	3+4=7	22.09	27.05	2.26	observation	NR	34	32.9	33
A-13	1, 56, 225	65	4+4=8	5.07	7.18	0.3	observation	NR	37.2	37.3	38.2

***partial responder - initial rapid decrease in PSA during the first four weeks, followed by an increase**
****sera samples not available for profiling at this timepoint**

B

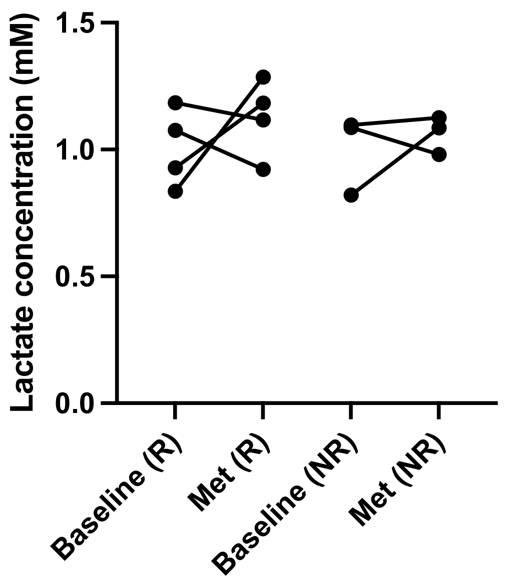

C

Patient Characteristics (n=25)	No metabolism-modifying drugs (15 patients)	On metabolism-modifying drugs (10 patients)
**Disease stage (% total patients)**	mHSPC/BCR: 53.3%<br>mCRPC: 46.7%	mHSPC/BCR: 60%<br>mCRPC: 40%
**Age (mean +/- SEM)**	70.7+/-1.7	71+/-2.5
**BMI (mean +/-SEM)**	25.6+/-1.1	31.7+/-1.5
**PSA (mean +/-SEM)**	59.05 +/- 31.1	2.1 +/- 0.87
**On ADT or ARSi**	12/15 (80%)	8/10 (80%)
**% of patients on metabolism-modifying drugs**	n/a	Metformin: 70%<br>Tirzepatide: 10%<br>Insulin: 10%<br>Semaglutide: 10%

A

Patient ID	WEIGHT (baseline, kg)	WEIGHT (56d, kg)	WEIGHT (225d, kg)
B-2	91.2	90.5	90.7
B-9	78.6	77.2	77.1
B-12	77.2	70	72.2
B-16	88.9	88.8	80.7
B-3	80.1	79.7	82.9
B-14	98.4	96.3	95.3
B-15	79	77.5	76.5
A-1	106.3	108.1	112.6
A-6	82.9	84.8	84.5
A-8	101.95	100.2	100.1
A-10	103.5	100.2	100.7
A-13	114.2	115.8	117.3

B

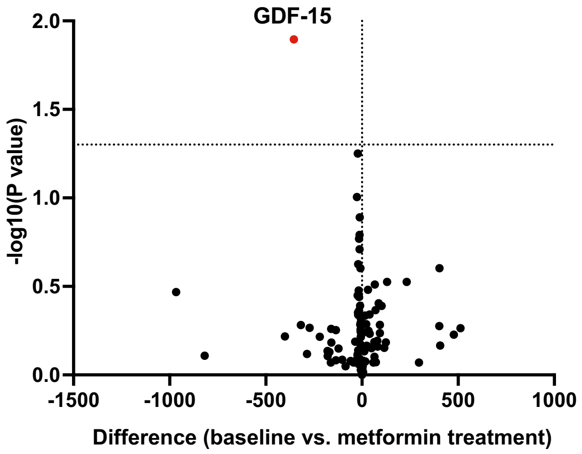

C

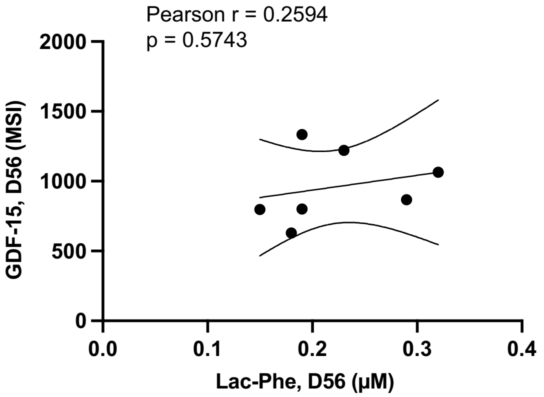

Figure EV2. GDF-15 is elevated by metformin treatment but does not significantly correlate with Lac-Phe levels.

(**A**) The weight of all patients ($n = 12$) at baseline (before treatment or observation), 2 months post-metformin treatment or observation (D56) and at the end of the trial (D225, including 6 months of metformin + bicalutamide, Met + Bic). (**B**) Volcano plot of significant changes in cytokine levels (mean signal intensity, MSI) following metformin monotherapy (D56), measured in Arm B patient sera. D56 values were compared to sera values at D1 and statistical significance determined via an unpaired Student's *t* test with Welch's correction for unequal variance. (**C**) Correlation between Lac-Phe levels (μM) vs. GDF-15 levels (MSI) at the D56 (metformin monotherapy) timepoint for Arm B patients. The linear regression line and 95% confidence intervals are shown, with Pearson correlation coefficient, *r*, and *P* value of the correlation noted above the graph. Source data are available online for this figure.

 © The Author(s)
